# Language Identification in Short Utterances Using Long Short-Term Memory (LSTM) Recurrent Neural Networks

**DOI:** 10.1371/journal.pone.0146917

**Published:** 2016-01-29

**Authors:** Ruben Zazo, Alicia Lozano-Diez, Javier Gonzalez-Dominguez, Doroteo T. Toledano, Joaquin Gonzalez-Rodriguez

**Affiliations:** ATVS-Biometric Recognition Group, Universidad Autonoma de Madrid, Madrid, Spain; University of Kent, UNITED KINGDOM

## Abstract

Long Short Term Memory (LSTM) Recurrent Neural Networks (RNNs) have recently outperformed other state-of-the-art approaches, such as i-vector and Deep Neural Networks (DNNs), in automatic Language Identification (LID), particularly when dealing with very short utterances (∼3s). In this contribution we present an open-source, end-to-end, LSTM RNN system running on limited computational resources (a single GPU) that outperforms a reference i-vector system on a subset of the NIST Language Recognition Evaluation (8 target languages, 3s task) by up to a 26%. This result is in line with previously published research using proprietary LSTM implementations and huge computational resources, which made these former results hardly reproducible. Further, we extend those previous experiments modeling unseen languages (out of set, OOS, modeling), which is crucial in real applications. Results show that a LSTM RNN with OOS modeling is able to detect these languages and generalizes robustly to unseen OOS languages. Finally, we also analyze the effect of even more limited test data (from 2.25s to 0.1s) proving that with as little as 0.5s an accuracy of over 50% can be achieved.

## Introduction

Language identification (LID) aims to automatically determine which language is being spoken in a given segment of a speech utterance [1]. In a globalized world where the use of voice-operated systems is more common every day, LID typically acts as a pre-processing stage for both human listeners (i.e. call routing to a proper human operator) and machine systems (i.e. multilingual speech processing systems) [2]. In most of these applications LID is used in real-time scenarios so computational cost is often critical.

Currently, most of the state-of-the-art systems for LID rely on acoustic modelling [3, 4]. Driven by recent developments in speaker verification, the basic approach of these systems involves using i-vector front-end features followed by a classification stage that compensates speaker and session variabilities [5–7]. An i-vector is a fixed-size representation (typically from 400 to 600 dimensions) of a whole utterance, derived as a point estimate of the latent variables in a factor analysis model [8, 9]. As an example of how common i-vectors are in the field, there will be a LID evaluation in 2015 organized by the Institute of Standards and Technology of America (NIST) where the data given are i-vectors instead of raw audio (thus, they call it *i-vector challenge*). Even though the i-vector approach has proven to be successful in several scenarios, including rapid LID [10], it suffers from two major drawbacks. First, the estimation of the point has a larger variance when the amount of data used to compute it decreases, quickly degrading its robustness, specially from short segments. Second, the i-vector is a compact representation of a whole utterance, so most of the computation is performed after completion of it, introducing a significant latency.

Recently, new approaches such as Deep feed forward Neural Networks (DNNs) have shown to outperform the previous dominant paradigms in different and varied machine learning applications—including acoustic modelling [11] and image classification [12] among others [13]. We have previously shown that DNNs also surpass the performance of i-vector based approaches in language recognition when enough data for training is available (≥20h per language); specially when dealing with short test utterances (≤3s) [14, 15].

The LID task in the NIST evaluations or in the Robust Automatic Transcription of Speech (RATS) projects provide a great framework to test and compare these new approaches. DNNs have been applied successfully to LID in three different ways:

1. Train a DNN or Convolutional Neural Network (CNN) with a bottleneck architecture and then use the outputs of the bottleneck layer as features within an i-vector framework [16–18].
2. Modify the standard i-vector technique to use senones predicted with a DNN trained for speech recognition as classes instead of the Gaussian components defined by a Gaussian mixture model (GMM) based universal background model (UBM) [17, 19].
3. Train a neural network in an end-to-end fashion to discriminate between languages [14].

However, while DNN-based approaches have proven to perform great in a variety of scenarios, they rely on stacking several acoustic frames as an input in order to model longer time context than a frame [14]. On the other hand, recurrent neural networks (RNNs), a special type of DNNs where connections between units form directed cycles, seem to be a more proper model when coping with time depending sequences [20]. Even though RNNs are, in theory, a good model to fit temporal sequences such as speech, its training process has issues that makes its performance not as good as expected [21, 22].

Long Short-Term Memory (LSTM) recurrent neural networks (RNNs) have the ability to store information from previous inputs during long time periods [23–25]; which makes them much more suitable to model sequential data than deep feed forward neural networks. LSTM RNNs have recently been shown to outperform the state of the art DNN systems in tasks involving time-depending signals including acoustic modeling in large vocabulary speech recognition [26] or handwriting recognition [27].

Motivated by those results we previously introduced a LID system based on LSTM RNNs with an outstanding performance in Gonzalez-Dominguez et al. (2014). So far, the solution implemented runs over a large machine infrastructure and includes a proprietary LSTM RNNs implementation. This fact makes its use hardly reproducible or simply inaccessible for many research groups.

In the current study, we adapt that approach in order to build an efficient system using open source software. Therefore, we explore different aspects that affect LSTM RNNs performance, implementing several configurations and comparing the obtained results with a reference i-vector based system trained from the same acoustic features. We also analyze this kind of systems when coping with out-of-set data or shorter durations (up to 0.1s). The results of our experiments show that a LSTM RNN system with an open-source implementation can perform better than our reference i-vector system.

The rest of this paper is organized into the following sections. Section [Reference System: i-vector] defines a reference system based on i-vectors. The LSTM RNN architecture is presented in Section [LSTM RNN as a language identification system]. The experimental protocol and datasets are described in section [Datasets and Evaluation Metrics]. Next, we examine the behavior of our scheme over a range of configuration parameters in both the task and the neural network topology. Finally, Section [Conclusions] is devoted to present the conclusions of the study and potential future work.

## Reference System: i-vector

Currently, state-of-the-art LID is performed relying on i-vector technology [8]. The use of i-vector as a front-end is so extended that NIST has recently organized an evaluation called *i-vector challenge 2015* where LID is perform given i-vectors instead of audio. Even though all such approaches share the i-vectors as a feature extraction or representation they differ in the kind of classifier used to perform the language identification [6]. In the rest of this Section, we describe: a) the i-vector extraction procedure, b) the i-vector classifier used in this study, and c) the configuration details of our reference i-vector system. This system will serve us as reference system to be compared with our LSTM RNN system.

### I-vector extraction

Driven by developments in speaker verification based on the MAP adaptation approach in a GMM framework [28], utterances in language identification are typically represented by the accumulated zero- and centered first-order Baum-Welch statistics, *N* and *F*, respectively, computed from an Universal Background Model (UBM), *λ*. For UBM mixture *m* ∈ 1, …, *C*, with mean, *μ*_*m*_, the corresponding zero- and centered first-order statistics are aggregated over all frames of the utterance as

$$N_{m}=\sum_{t}p\left(m|o_{t},\lambda \right)$$ (1)

$$F_{m}=\sum_{t}p\left(m|o_{t},\lambda \right)\left(o_{t}-\mu_{m}\right),$$ (2)

where *p*(*m*|*o*_*t*_, *λ*) is the Gaussian occupation probability for the mixture *m* given the spectral feature observation *o*_*t*_ ∈ ℜ^*D*^ at time *t*.

Intuitively, the Total Variability (TV) model reduces the dimension of the *supervector* constraining all the variability into the *i-vector*. It can be seen as a classical Factor Analysis (FA) generative model [29] with

1. The vector of observed variables, *supervector* (CD x 1), formed by the statistics stacked *F* = {*F*_1_, *F*_2_, …, *F*_*C*_}. (CD ∼ 10k–100k)
2. The vector of hidden variables, i-vector, *w* ∈ ℜ^*L*^. (L ∼ 200–800)
3. The rectangular low rank matrix *T* ∈ ℜ^*CD*×*L*^, which relates observed and hidden variables

$$N^{-1}F=Tw,$$ (3)

where the zero-order statistics *N* are represented by a block diagonal matrix ∈ℜ^*CD*×*CD*^, with *C* diagonal *D* × *D* blocks. The *m*-th component block is the matrix *N*_*m*_ *I*_(*D*×*D*)_. Given the imposed Gaussian distributions of *p*(*w*) and *p*(*F*|*w*), it can be seen that the mean of the posterior *p*(*w*|*F*) is given by

$$w={\left(I+T^{t}\Sigma^{-1}NT\right)}^{-1}T^{t}\Sigma^{-1}F,$$ (4)

where Σ ∈ ℜ^*CD*×*CD*^ is the diagonal covariance matrix of *F*. The TV model is thus a data driven model with parameters {*λ*, *T*, Σ}. A more detailed explanation of the derivation of these parameters using the EM algorithm can be found in [9].

### Classification

In the TV model, *T* constrains all the variability (i.e. language, speaker, session) and it is shared for all the language models. Therefore, the i-vectors, *w*, can be seen as a new representation of the input to classify. Further, several classifiers can be used to perform classification [6].

In Speaker Recognition (SR), dimensionality reduction of the i-vector by Linear Discriminant Analysis (LDA) before applying the scoring technique significantly improves the performance. However, it was found that LDA is not that convenient in LID because it projects the i-vectors into a (N-1)-dimensions space, where N is the number of target languages, loosing relevant information for LID [30]. For our experiments we found that applying dimensionality reduction by LDA or the PLDA model did not improve the performance. Accordingly, Cosine Distance scoring without LDA, which performed best, has been used. Thus, the similarity measure (score) for a given test utterance i-vector *w*, and the mean i-vector *w*_*L*_ of the language *L* is given by

$$S_{\left(w,w_{L}\right)}=\frac{\left\langle w,w_{L}\right\rangle }{\left||w|||\right|w_{L}\left|\right|}$$ (5)

### Feature extraction and configuration parameters

As input features for this study we used Mel Frequency Cepstral Coefficients with Shifted Delta Coefficients (MFCC-SDC) with the configuration 7-1-3-7 extracted with a 10ms frame shift from 20ms windows (each frame forms a vector input of dimension 56). From those features, we built a UBM of 1024 components. The TV matrix was derived using PCA and a posterior refinement of 5 EM iterations [31], keeping just the top 400 eigenvectors. Finally we derived the i-vectors using the methodology presented in Section [I-vector extraction]. In addition, silence frames were filtered out using a voice activity detector based on energy.

The total number of parameters of the i-vector system accounts for size of the TV matrix. It is given by *N*x*D*x*F*, being *N*, *F* and *D* the number of Gaussians components (1024), the i-vector dimensions (400) and the feature dimension (56) respectively. In our model, this makes a total of ∼23M of parameters.

## LSTM RNN as a language identification system

**Fig 1:**
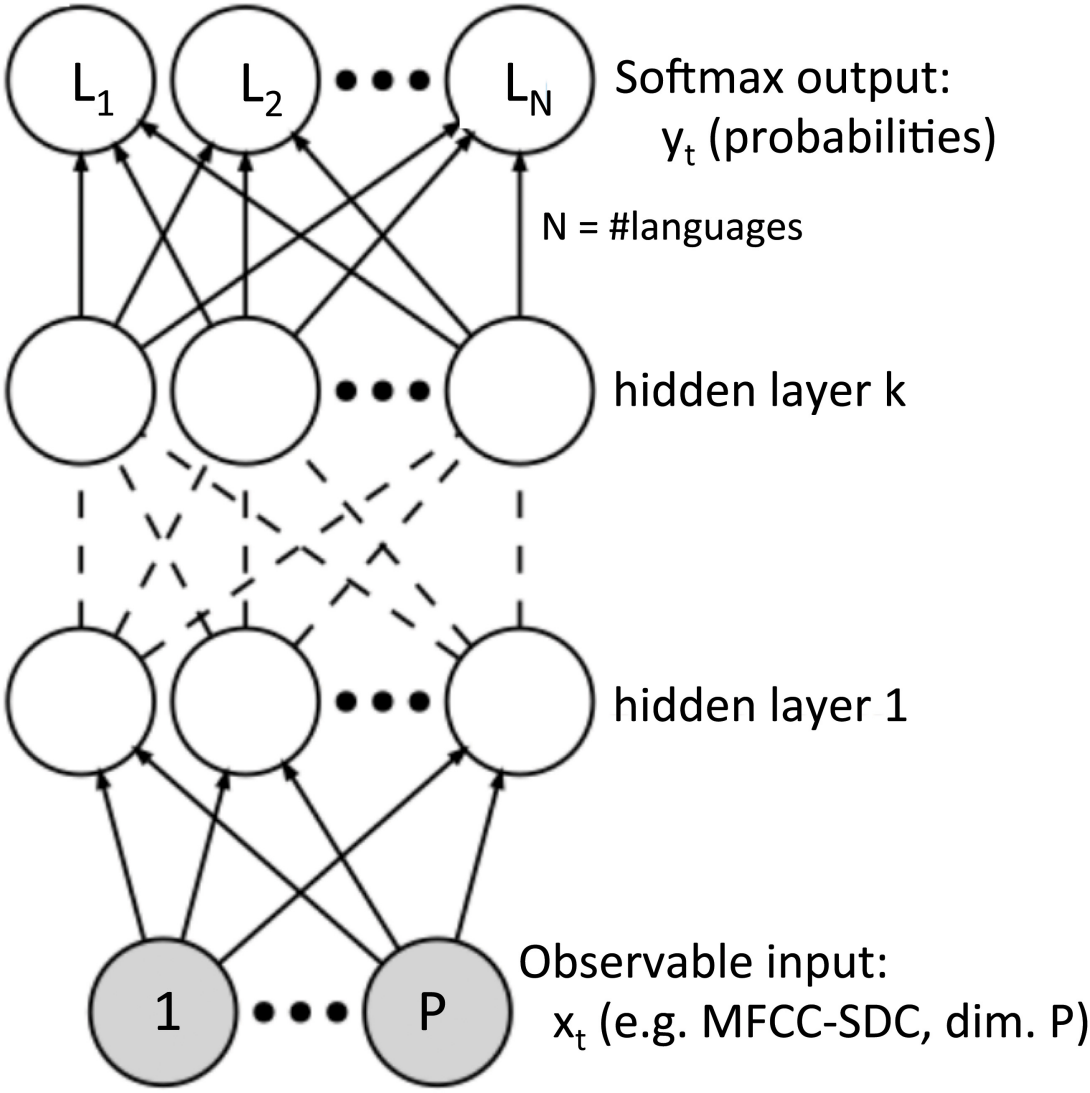
DNN network topology. In this paper we use one or two hidden layers replacing each hidden unit with an uni-directional LSTM memory block with forget gates and peepholes.

It was shown recently in the field of LID, among others, that significant performance improvements over classical GMM-based systems can be achieved through the use of deep neural networks [32]. In Fig 1 the topology of a standard fully-connected DNN is shown. Among the most important advantages of DNNs is their multilevel distributed representation of the input [11]. Also DNNs do not require detailed assumptions about the input data distribution [33] and have proven to be successful exploiting large amounts of data without lapsing into overtraining. On the other hand, DNNs rely on stacking several acoustic frames as an input in order to model longer time context than a frame and have no ability to model sequences in a proper way [14].

Recurrent Neural Networks (RNNs) are a DNN where connections between units form directed cycles. They seem a very powerful tool to model sequences because they are not limited to discrete state spaces. RNNs use the internal state as a memory for mapping real-valued input sequences to real-valued output sequences exhibiting dynamic temporal behavior. This fact makes them a more proper way to model temporal sequences [20]. However, classical RNNs have been plagued by a major practical problem: the gradient of the total output error with respect to previous inputs quicky vanishes as the time lags between relevant inputs, known as *vanishing gradient problem* [21, 22]. Hence, classical RNNs fail to learn when time lags exceeds more than 5–10 discrete time steps between relevant inputs and target signals [34].

**Fig 2:**
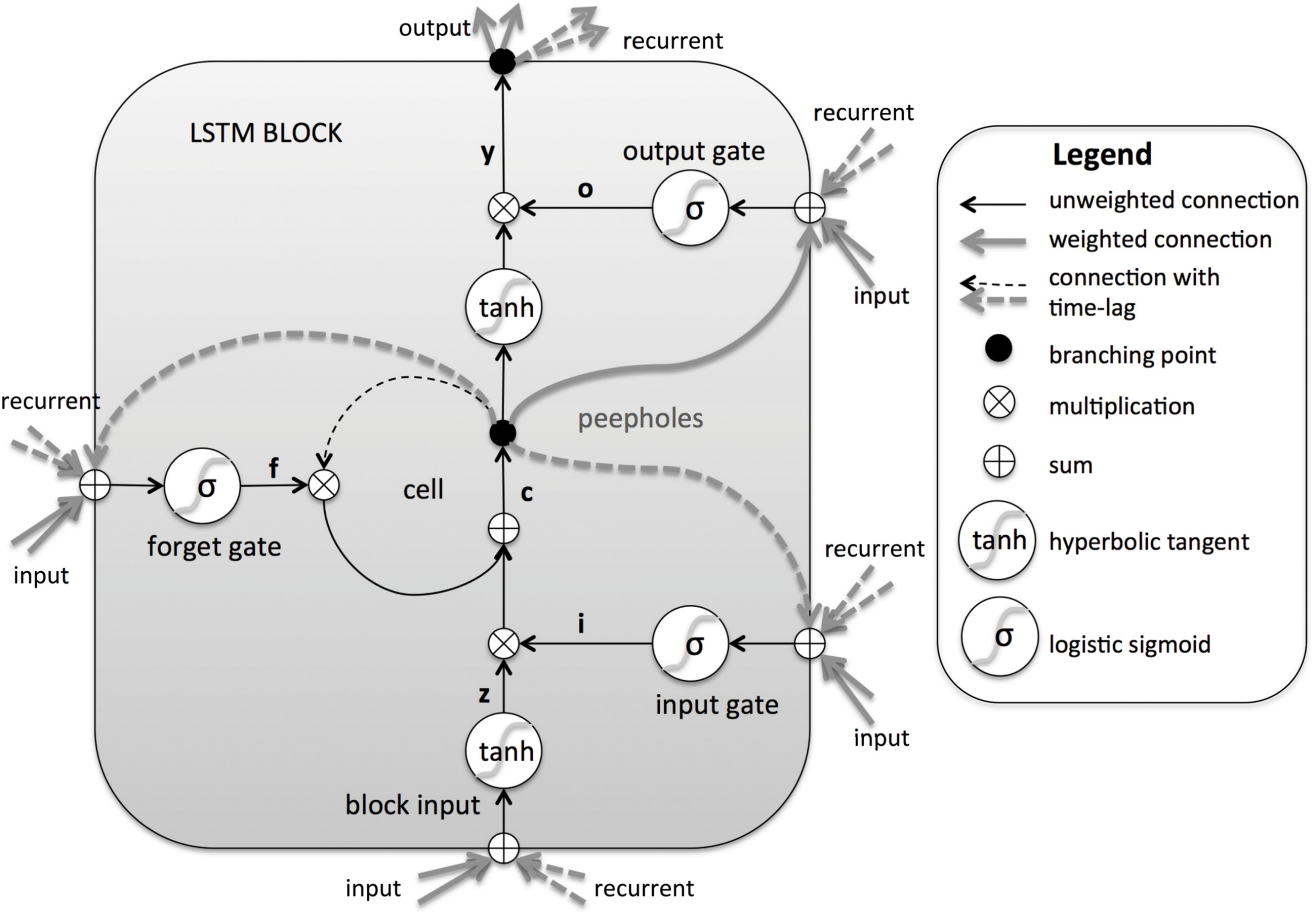
Long Short-Term Memory recurrent neural network architecture. A single memory block is shown for clarity. The output goes to every unit in the next layer. The recurrent output goes to this memory block and every other memory block in this layer. All inputs and recurrent inputs shown are the same signals (same input goes to the memory block and to the three gates). Adapted from [36].

The Long Short Term Memory architecture [24, 35] was motivated by these drawbacks. The underlying idea of a LSTM neural network is to replace the hidden units in a traditional DNN with *memory blocks* [36] (see Fig 2). These blocks can be seen as a memory chip in a digital computer; each one contains one or more memory cells recurrently connected and three multiplicative units -the input, output and forget gates- that work similar to the write, read and reset signals in that chip. More precisely, the input and output gates control respectively the flow of input activations into the memory cell and the output flow of cell activations into the rest of the network. The forget gate allows the flow of information from the memory block to the cell as an additive input, therefore adaptively forgetting or resetting the cell’s memory. These features make them easier to train properly than conventional RNNs. The input and output gates help solving the vanishing error problem in the traditional RNN [21]: in the absence of a new input or error signals to the cell, the local error remains constant. In addition, the forget gate allows the network to have an adaptive and limited memory buffer avoiding infinite loops. The LSTM architecture described in [25] also has the ability to learn precise timing of the outputs using *peephole* connections. These connections allow communication between gates of the same memory block; outperforming the traditional architecture specially when precise timing of the outputs is important.

### Architecture

A schematic of a unit of the LSTM RNN used in this work can be seen in Fig 2. It features a single memory cell, three gates (input, forget and output), an output activation function and peephole connections. The output of the block is recurrently connected to the block input and all of the gates.

The vector formulas for a LSTM layer forward pass are given below. More details and the corresponding Back Propagation Through Time (BPTT) formulae can be found in [37]. Here *x*^*t*^ is the input vector at time *t*, the *W* are rectangular input weight matrices, the *R* are square recurrent weight matrices, the *p* are peepholes weight vectors and *b* are bias vectors. *σ* is the logistic sigmoid function, *tanh* is the hyperbolic tangent activation function and ⊙ is the element-wise product of the vectors.

$$\begin{array}{c} z^{t}=tanh\left(W_{z}x^{t}+R_{z}y^{t-1}+b_{z}\right)\;\;\;\;\text{block input}\; \end{array}$$ (6)

$$i^{t}=\sigma (W_{i}x^{t}+R_{i}y^{t-1}+p_{i}⊙c^{t-1}+b_{i})\;\;\;\;\text{input gate}$$ (7)

$$f^{t}=\sigma (W_{f}x^{t}+R_{f}y^{t-1}+p_{f}⊙c^{t-1}+b_{f})\;\;\;\;\text{forget gate}$$ (8)

$$\begin{array}{c} c^{t}=i^{t}⊙z^{t}+f^{t}⊙c^{t-1}\;\;\;\;\text{cell state} \end{array}$$ (9)

$$o^{t}=\sigma (W_{o}x^{t}+R_{o}y^{t-1}+p_{o}⊙c^{t}+b_{o})\;\;\;\;\text{output gate}$$ (10)

$$\begin{array}{c} y^{t}=o^{t}⊙tanh\left(c^{t}\right)\;\;\;\;\text{block output} \end{array}$$ (11)

The output layer is then configured as a *softmax*, where hidden units map input *x*_*j*_ to a class probability *p*_*j*_ in the form

$$p_{j}=\frac{exp(x_{j})}{\sum_{l}exp\left(x_{l}\right)}$$ (12)

where *l* is an index over all the classes.

As a cost function for backpropagating gradients in the training stage, we use the cross-entropy function defined as

$$C=-\sum_{j}t_{j}\log p_{j}$$ (13)

where *t*_*j*_ represents the target probability of the class *j* for the current evaluated example, taking a value of either 1 (true class) or 0 (other class).

### System description

In this work we present an efficient LSTM based system for LID built from the conceptual architecture explained above. The system described runs on a single Tesla K20 GPU with 4 Gb of memory. The software used to build the LSTM neural networks is CURRENNT [38]; a machine learning library which implements uni-directional and bi-directional LSTM architectures using NVIDIA graphic cards to accelerate the computations. More details about the software can be found in [39].

As input to the net we used the same acoustic features used for the i-vector reference system; MFCC-SDC with the configuration 7-1-3-7 extracted with a 10ms frame shift from 20ms windows using Kaldi [40] (input vector of dimension 56). As we are coping with short test utterances, in order to train the network in similar conditions, the development dataset has been split into random chunks of 2 seconds. From the development dataset, defined in Section [Datasets and Evaluation Metrics], we take 85% of the files to train the net and the other 15% will be used as a validation set for early stopping and selection of the best model. Specifically, the input layer is fed with no stacking of acoustic frames: a single MFCC-SDC is given as an input at a time-step. Thus, the input layer has a total number of 56 visible units.

On top of the input layer, our systems consist of one or two hidden layers followed by an output layer. The hidden layers are uni-directional LSTM layers with forget gates and peepholes and the number of hidden layers contained ranges from 256 to 1024 LSTM memory cells. Then, we added the softmax layer with the same number of units as classes we have in our experiment. We will distinguish between our standard LSTM RNN system, with the same outputs as target languages we have and our out of set LSTM RNN system, which has 1 more output that target languages in order to model an out of set class. The softmax layer utilizes a cross entropy error function for the back propagation and returns a probability for each input frame and language. To train the network we are using stochastic gradient descent with 100 parallel sequences at the same time and a learning rate of 2e-07. Therefore, the trainer updates the network weights after every processed fraction of parallel sequences.

In the LSTM hidden layers, the memory blocks store the temporal state of the network acting as a memory which changes with the input to the neural network at each time step. The softmax output for a given frame is a probability of belonging to one of the languages as an output, but it relies not only on the frame input but on every previous frame in that sequence or file. Therefore, the system decides every output based on previous and present inputs. Thus, the last outputs are the most reliable because they are computed when the system has information of almost the whole file. For scoring, we compute an utterance level score for each target language by averaging the log of the softmax output for that language but taking into account just the last ten percent of the total frame scores for every file.

## Datasets and Evaluation Metrics

We have conducted our experiments following the well-known protocol provided by the USA National Institute of Standards and Technology (NIST) Language Recognition Evaluation (LRE) 2009 framework, LRE’09 from now on. This framework allows us to evaluate the proposed methods with a large collection of data and provides a reproducible benchmark comparable with other related work in the field.

### The NIST Language Recognition Evaluation 2009 dataset

The Language Recognition Evaluation organized by NIST consisted, for the first time in 2009, in a mixture of two types of data:

- Conversational Telephone Speech (CTS), used in the previous evaluations for both development and test purposes, consisting of spontaneous telephone conversations.
- Broadcast news data from “Voice of America” (VOA), obtained via an automatic acquisition system where telephone and non-telephone speech are mixed.

Up to 2TB of 8KHz raw data containing radio broadcast speech, with the corresponding language and audio source labels were distributed to participants; a total of 40 languages (23 target and 17 out of set) were included.

All the data considered in our experiments belongs to VOA in order to avoid unbalanced mix of CTS and VOA. Further, we have selected 8 representative languages for which up to 200 hours of audio are available, as it was already done in [41], in an effort to avoid the disparity on training data for every language (from ∼10 to ∼950 hours). We have used all the other target languages in LRE’09 to build a train set with ∼200 hours in order to train an out of set class. Thus, we differentiate 3 different sets of languages in our experiments:

- Target languages: US English (eng), Spanish (spa), Dari (dar), French (fre), Pashto (pas), Russian (rus), Urdu (urd) and Chinese Mandarin (chi).
- Trained out of set languages (trained_OOS): This set contains all the other target languages considered in LRE’09 (more details given in Table 1).
- Real out of set languages (real_OOS): This set contains all the out of set languages considered in LRE’09 (more details given in Table 1).

**Table 1:** List of languages. List of all the different language sets considered in our experiments.

Target languages	Trained_OOS	Real_OOS
US_English (eng)	Amharic	Arabic
Spanish (spa)	Bosnian	Azerbaijani
Dari (dar)	Cantonese	Belorussian
French (fre)	Haitian Creole	Bengali
Pashto (pas)	Croatian	Bulgarian
Russian (rus)	Indian English	Unknown English
Urdu (urd)	Farsi	Italian
Mandarin Chinese (chi)	Georgian	Japanese
	Hausa	Punjabi
	Hindi	Romanian
	Korean	Shanghai-wu
	Portuguese	Southern-min
	Turkish	Swahili
	Ukrainian	Tagalog
	Vietnamese	Thai
		Tibetan
		Uzbek

Our test sets are the following:

- **main_test_set**: consists of the trials from the NIST LRE’09 3s condition evaluation set belonging to VOA for the 8 target languages, yielding a total of 2942 test segments and 23536 trials.
- **trained_OOS_test_set**: formed by the main test set plus all the VOA trials of the 3s condition in LRE’09 of the trained_OOS languages (see Table 1) leading to 8931 (where 5989 of them are oos) test segments and a total of 80379 trials.
- **real_OOS_test_set**: constrains the main test set plus all the VOA trials of the 3s condition in LRE’09 of the real_OOS languages (see Table 1) yielding to 6437 test segments (where 3495 of them oos) test segments and a total of 57933 trials.
- **cts_test_set**: consists of the trials from the NIST LRE’09 3s condition evaluation set belonging to CTS. From the 8 target languages only 4 have CTS test files (chi, rus, eng and urd) yielding a total of 1320 test segments and 10560 trials.

In addition, we wanted to evaluate the performance of our system when even shorter test utterances are considered. Therefore, we have selected all the files in our main test set with more than 2.25 seconds of speech according to our VAD yielding to a subset of 2100 files. Then, we have cut these recordings to build different duration subsets ranging from 0.1 to 2.25 seconds of speech.

### Evaluation Metrics

Two different metrics were used in order to assess the performance of our systems:

- Accuracy. The % of correctly identified trials when making hard decisions (by selecting the top scored language).
- *EER*_*avg*_, the mean of the Equal Error Rate computed language by language which is an extended metric in the community.

For the sake of clarity we do not deal with the problem of setting optimal thresholds (calibration). Therefore, *C*_*avg*_, the metric used in the LRE’09 evaluation is not used in this work.

## Results and Discussion

### System performance

In [41] it was shown that LSTM RNNs are a good approach to exploit useful temporal information for LID with proprietary implementation. Motivated by those results, in this study we explore different architectures and configurations using open-source code.

Table 2 summarizes the performance of 5 LSTM RNNs systems in terms of *EER*_*avg*_ and accuracy. We highlight first that 4 out of the 5 proposed architectures for the LSTM RNN system outperform the reference i-vector based system in *EER*_*avg*_. This fact is particularly interesting taking into account that the proposed architectures have from 5 to 21 times fewer parameters (see *Size* in Table 2) than the reference system.

**Table 2:** System performance on the 8 target languages subset of LRE’09 (3s test segments). The last column stands for the improvement in terms of *EER*_*avg*_ with respect to the reference system.

**Architecture**			**Complexity**		**Performance**		
ID		Size	Time / Iter	#Iters	Accuracy(%)	*EER*_*avg*_(%)	Improvement (%)
#1	reference i-vector based system	∼23M			65.02	16.94	-
#2	lstm_1_layer_512_units	∼1.2M	∼25 min	∼125	57.51	17.82	-
#3	lstm_1_layer_750_units	∼2.5M	∼45 min	∼300	63.39	15.61	∼7.85
#4	lstm_1_layer_1024_units	∼4.4M	∼75 min	∼350	65.63	15.10	∼10.86
#5	lstm_2_layer_256_units	∼850k	∼20 min	∼500	63.73	14.96	∼11.69
#6	lstm_2_layer_512_units	∼3.3M	∼60 min	∼400	**70.90**	**12.51**	**∼26.15**

Table 2 also shows the computational complexity of the different topologies in terms of time per training iteration and the approximate number of iterations necessary until convergence. Even though the number of iterations depends on many factors (initialization, learning rate, etc.) we can observe that both the time per iteration and the number of iterations increase with the total size of the model.

**Fig 3:**
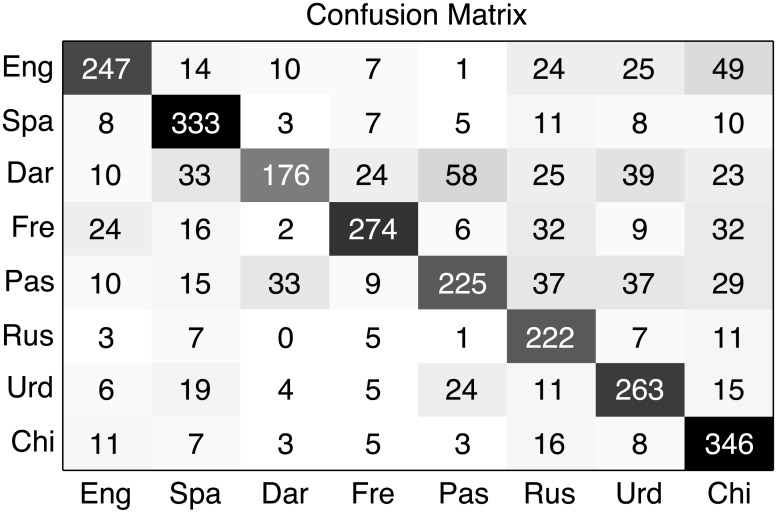
Best system confusion matrix. Confusion matrix of the best LSTM RNN single system, lstm_2_layer_512_units (system #6), on the 8 target languages subset of LRE’09 (3s test segments). Ground truth is represented in the Y axis while the predicted language is represented in the X axis.

While *EER* is widely used in the field of LID, in this experiment we are facing a multi-class classification problem. In order to analyze both the confusion and the discrimination performance of the systems considering all the languages pairs, Fig 3 shows the confusion matrix of the best single system, #6 in Table 2. In this confusion matrix we can see that our system predicts correctly most of the test segments but there are still some frequent confusions as Dari—Pashto.

### Out of set performance

In this section we want to show the performance of LSTM RNN based systems for language identification in presence of out of set (OOS) languages. In order to face this problem we have trained an additional OOS class with a mixture of audio coming from 15 different languages (see Table 1 for more details). We have selected three systems for comparison; the reference i-vector based system, the single lstm system with best performance so far (512 units per layer and 2 layers), and the same system with a single layer (512 units per layer and 1 layer) to test this task. Results are summarized in Table 3.

**Table 3:** Out of set system performance. The two first systems are the same than shown in Section [System performance]. The two systems following are systems capable of dealing with out of set task.

	Average *EER*(%)		
	main_test_set	trained_oos_test_set	real_oos_test_set
reference i-vector based system	16.94	21.60	21.87
lstm_1_layer_512_units	17.82	-	-
lstm_2_layer_512_units	**12.51**	-	-
oos_lstm_1_layer_512_units	18.14	23.57	23.75
oos_lstm_2_layer_512_units	14.83	**20.84**	**21.10**

We highlight three major results. First, when dealing with the main test set (no out of set test data) the performance of the oos LSTM RNN system (the system trained with 1 class for modeling out of set inputs) degrades moderately with respect to the standard system. This result was expected because the new class we are training is harder to learn than the 8 initial classes (it is seeing utterances from 15 languages instead of just one). Thus, the error, when back propagated, dominates the network at training time disturbing the fine-tune of the classes corresponding the 8 target languages. Moreover, among the languages used to train the OOS class, there are languages highly similar with the target languages (e.g. Indian English vs U.S. English). Second, the performance when dealing with real out of set data is as good as it is when dealing with the trained out of set dataset. Having comparable performance with those datasets proves the potential ability of this kind of neural network to tackle the out of set problem (network fed with unseen classes as inputs) and generalize robustly. Finally, we can see that the LSTM system performs better than the reference system, proving its robustness, but the gap gets much closer when dealing with out of set data.

**Fig 4:**
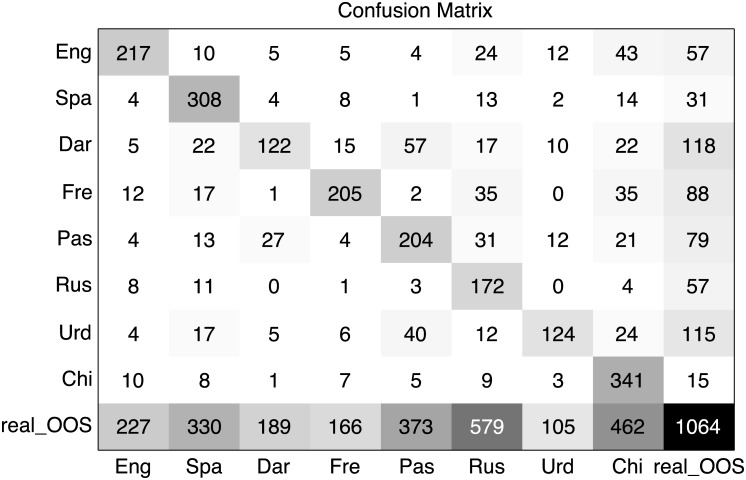
Out of set confusion matrix. Confusion matrix of the best out of set LSTM RNN system, lstm_2_layer_512_units (system #6), on the real out of set LRE’09 set (3s test segments). Ground truth is represented in the Y axis while the predicted language is represented in the X axis.

In order to have a better insight into the behavior of the LSTM RNN system when dealing with out of set test segments, we show in Fig 4 the confusion matrix of the best out of set system, oos_lstm_2_layer_512_units, when fed with real out of set test utterances. The most important point here is to see how, while not disturbing too much the classification of the 8 target languages (compare with Fig 3), the accuracy obtained for the out of set test segments is far worse. Thus, while our approach seems a good starting point there is still room for further improvement.

### Limited duration test utterances

In this section we show the results obtained with even shorter versions of our test set in order to gain some insight about the relation between the performance of the system (in terms of accuracy) and the length of the test utterances. Thus, in this Section we explore the performance degradation when limiting the test duration. Following the same reasoning than in Section [Out of set performance], the systems used to perform this task are the systems with 512 units per layer and one or two layers.

**Fig 5:**
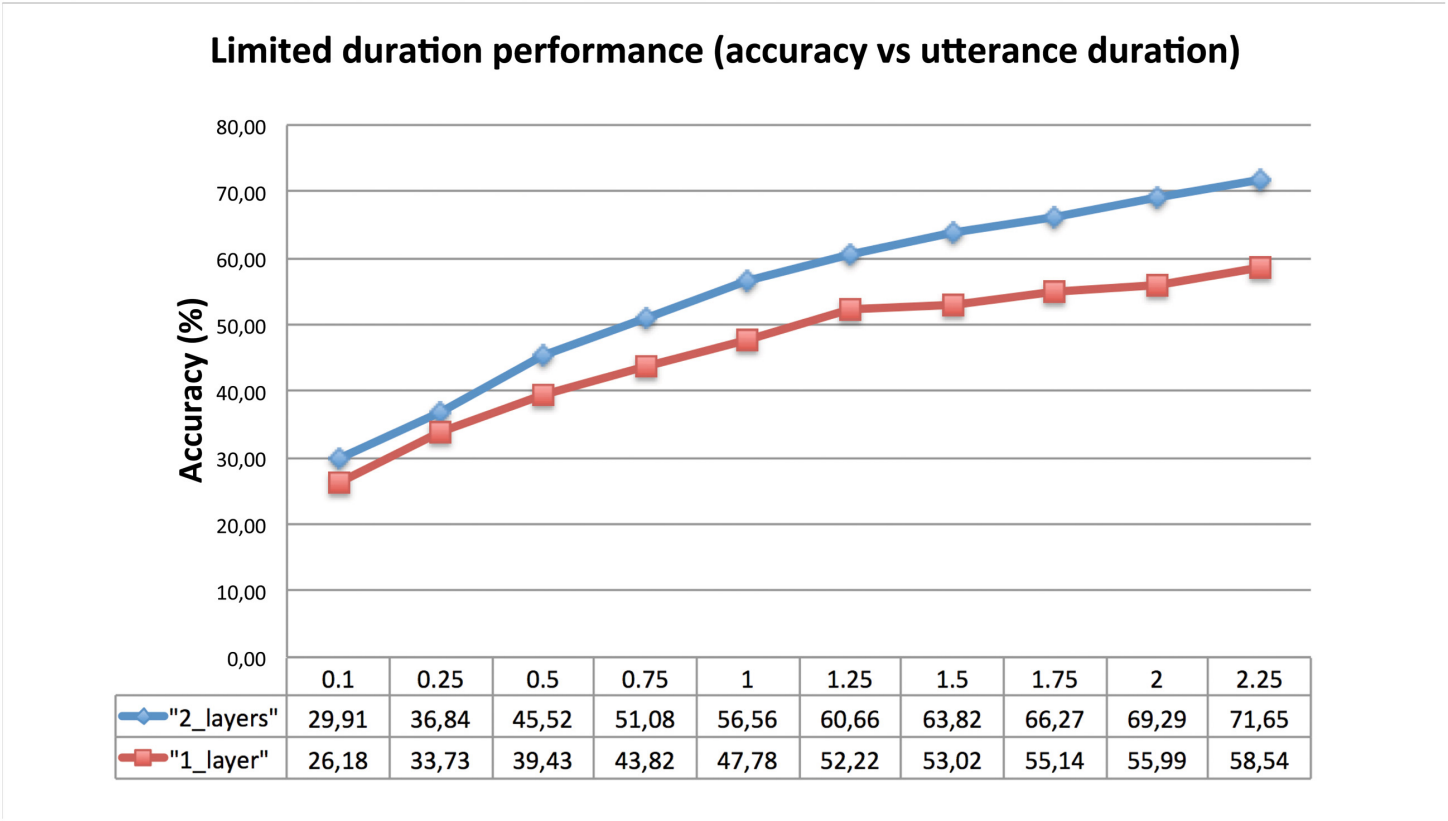
Limited duration performance. Accuracy of our LSTM RNN systems when dealing with fixed-time, super-short test utterances.

Fig 5 shows the average accuracy as a function of the test duration (more details can be found in Section [Datasets and Evaluation Metrics]). To have a better understanding on those results it may be useful to realize that a typical phoneme duration is about 60–70 ms so we would like to highlight that accuracy rates about 30% are achieved with less than two phonemes in average. Second, as expected, the performance of both systems increase with the duration of the test so a more reliable system can be built when larger test utterances are present.

### Testing with CTS data

In this paper we chose our data to be VOA only (as we already did in our previous work) in order to avoid effects due to a mismatch or imbalance between different databases. In this section, we want to test how our system performs in presence of CTS data, even though our neural networks and i-vector systems have been trained only on VOA data. The systems selected for this experiment are, for the same reasons, the ones chosen for the out-of-set experiments. In Table 4 we can see that the LSTM system performs even better with CTS data and clearly outperforms the i-vector based system, proving its robustness and generalization capabilities.

**Table 4:** Mismatched performance. All the systems shown here have been trained only with VOA audio while tested on both VOA and CTS data.

	Average *EER*(%)	
	main_test_set	cts_test_set
reference i-vector based system	16.94	25.17
lstm_1_layer_512_units	17.82	16.09
lstm_2_layer_512_units	**12.51**	10.71

## Conclusions

In this work, we present an analysis of the use of Long Short Term Memory (LSTM) Recurrent Neural Networks (RNNs) for Automatic Language Identification (LID) of short utterances. Motivated by the recent success of LSTM RNNs with proprietary LSTM RNNs implementation when large-infrastructure is available, we aimed to implement and extend the insights of that work using open source software and much more limited resources.

Through this study, we explore several configurations of LSTM RNNs systems and compare them with an i-vector based system. Results on a subset of NIST LRE 2009 (8 languages selected and a 3s VOA condition) show that LSTM RNN based systems achieve better performance than the reference i-vector system using fewer parameters (∼1–5M vs ∼23M).

In addition, we have evaluated the accuracy of the system when using very short utterances (from 0.1 to 2.25 seconds according to our VAD). We find, for our proposed LSTM RNN scheme, that accuracy over 70% may be achieved with about 2 seconds while more than 0.5 seconds are needed in order to obtain an accuracy rate over 50%. Further, we have tackled the problem of non seen languages (out of set) with an LSTM RNN approach with little degradation when dealing with out of set languages with respect to the system tested with the languages used to train the out of set class. Finally, we have tested our systems with a mismatch in the databases (training with VOA and testing with CTS) obtaining results comparable with the matched experiment, the proposed system outperforms the reference i-vector system on the same conditions, proving its robustness.

Results using NIST LRE 2009 (8 languages selected) demonstrate that LSTM RNN based approaches using open source implementations outperform the standard i-vector based approach when dealing with short test durations with an improvement of ∼26% in terms of Equal Error Rate with respect to the reference i-vector based system.
